# Probing Receptor Specificity by Sampling the Conformational Space of the Insulin-like Growth Factor II C-domain*[Fn FN2]

**DOI:** 10.1074/jbc.M116.741041

**Published:** 2016-08-10

**Authors:** Rozálie Hexnerová, Květoslava Křížková, Milan Fábry, Irena Sieglová, Kateřina Kedrová, Michaela Collinsová, Pavlína Ullrichová, Pavel Srb, Christopher Williams, Matthew P. Crump, Zdeněk Tošner, Jiří Jiráček, Václav Veverka, Lenka Žáková

**Affiliations:** From the ‡ Institute of Organic Chemistry and Biochemistry, Academy of Sciences of the Czech Republic, v.v.i., Flemingovo nám 2, 166 10 Prague 6, Czech Republic,; § Faculty of Science, Charles University in Prague, Albertov 6, Prague 128 43, Czech Republic,; ‖ Department of Analytical Chemistry, University of Chemistry and Technology, Technická 5, 166 28 Prague 6, Czech Republic,; ¶ Institute of Molecular Genetics, Academy of Sciences of the Czech Republic, v.v.i., Vídeňská 1083, 142 20 Prague 4, Czech Republic, and; ** Department of Organic and Biological Chemistry, School of Chemistry, Cantock's Close, University of Bristol, Bristol BS8 1TS, United Kingdom

**Keywords:** insulin, insulin receptor, insulin-like growth factor (IGF), nuclear magnetic resonance (NMR), structural biology, structure-function

## Abstract

Insulin and insulin-like growth factors I and II are closely related protein hormones. Their distinct evolution has resulted in different yet overlapping biological functions with insulin becoming a key regulator of metabolism, whereas insulin-like growth factors (IGF)-I/II are major growth factors. Insulin and IGFs cross-bind with different affinities to closely related insulin receptor isoforms A and B (IR-A and IR-B) and insulin-like growth factor type I receptor (IGF-1R). Identification of structural determinants in IGFs and insulin that trigger their specific signaling pathways is of increasing importance in designing receptor-specific analogs with potential therapeutic applications. Here, we developed a straightforward protocol for production of recombinant IGF-II and prepared six IGF-II analogs with IGF-I-like mutations. All modified molecules exhibit significantly reduced affinity toward IR-A, particularly the analogs with a Pro-Gln insertion in the C-domain. Moreover, one of the analogs has enhanced binding affinity for IGF-1R due to a synergistic effect of the Pro-Gln insertion and S29N point mutation. Consequently, this analog has almost a 10-fold higher IGF-1R/IR-A binding specificity in comparison with native IGF-II. The established IGF-II purification protocol allowed for cost-effective isotope labeling required for a detailed NMR structural characterization of IGF-II analogs that revealed a link between the altered binding behavior of selected analogs and conformational rearrangement of their C-domains.

## Introduction

The insulin-insulin-like growth factor (IGF)^4^ axis is a complex signaling pathway mediated by a group of three sequentially and structurally homologous peptide hormones, their membrane receptors, and several circulating IGF-binding proteins. Insulin and IGF-I and -II are all capable of higher or lower affinity binding toward the transmembrane tyrosine kinase receptors insulin receptor isoform A (IR-A), insulin receptor isoform B (IR-B), and insulin-like growth factor type I receptor (IGF-1R) (1, 2). All three receptors also share a high degree of homology, which is manifested by overlapping biological responses upon ligand binding (3–5). Binding of insulin and IGFs to the receptors triggers two major signaling pathways via autophosphorylation of tyrosines within their intracellular tyrosine kinase domains. The first, usually referred to as a phosphoinositide 3-kinase (PI3K)/Akt pathway, is key for the metabolic effects of ligand binding such as a decrease in plasma glucose levels (6). The second signaling pathway, referred to as Ras/ERK, involves activation of the Ras/Raf/MAPK/ERK1/2 cascade, which mediates proliferative effects through gene transcription regulation (7). Whereas insulin signals mainly via both IR isoforms (8), IGF-I and IGF-II promote the mitogenic signaling through IGF-1R (9, 10), and similar mitogenic stimulation results from IGF-II binding to IR-A (11).

Both IGFs are essential for embryonic development and are present in serum at nanomolar concentrations in adults (12) with IGF-II levels being 3-fold higher than IGF-I levels (13). Whereas the role of IGF-II in tumor development is well documented (14), its physiological role remains unclear. It is known that IGF-II is important for fetal development and placental function (15, 16), and several animal studies indicate an important role for IGF-II in memory enhancement (17–19). The availability of IGF ligands for signaling is modulated by a family of high affinity IGF-binding proteins 1–6 (20, 21) and insulin-like growth factor type II receptor (IGF-2R) (22). The equilibrium of individual components and the appropriate function of the entire insulin-IGF system are essential for biological responses such as regulation of basal metabolism, cellular growth, proliferation, survival, and migration (23).

**FIGURE 1.**
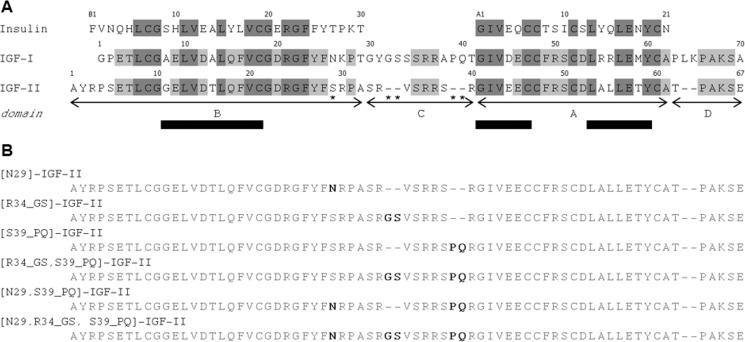
*A*, the amino acid sequence alignment of insulin, IGF-I, and IGF-II. It illustrates their high primary structure homology with the conserved residues highlighted in *dark gray* and the residues conserved between IGF-I and IGF-II in *light gray*. The organization of IGF-I and IGF-II into B-, C-, A-, and D-domains is shown below the sequences; domains A and B correspond to insulin A and B chains. The positions of conserved α-helices are shown as *bars* above the sequences. IGF-II residues mutated in this study are labeled with an *asterisk. B*, the amino acid sequence of the six prepared IGF-II analogs with highlighted mutations.

IGF-I and IGF-II are single chain peptides composed of 70 and 67 amino acids, respectively. Mature IGFs consist of four domains: B, C, A, and D in order from the N terminus. IGF-I and -II share over 60% sequence identity, mostly in the B- and A-domains that correspond to the B and A chains in insulin (Fig. 1). The 3D structure of IGF-I was obtained by both NMR and x-ray (24–34), whereas the structure of IGF-II has been determined only by NMR (35, 36). Together with insulin, these hormones share the insulin-like conformation consisting of three highly conserved α-helices (Fig. 1) further stabilized by three characteristic disulfide bonds (28, 36, 37).

IR-A, IR-B, and IGF-1R are homodimeric, and each monomer consists of an extracellular subunit (α) and transmembrane subunit (β) that are linked via four disulfide bonds into a functional β-α-α-β homodimer (38–40). The alternative splicing of IR exon 11 generates a 12-amino acid sequence in the C terminus of the α-subunit or IR-B that is absent in IR-A (41–43). Each monomer contains two insulin/IGF binding sites termed the primary (1) and second (2) site on one monomer and 1′ and 2′ on the partner. The primary binding site is formed from a leucine-rich repeat region (L1) and C-terminal helix (α-CT) region that combine with the second site of the partner monomer (2′) to form the complete binding pocket. The two sites (1-2′) bind a single molecule of insulin/IGF, triggering structural rearrangements and negative cooperativity for binding at the 1′-2 site (44–46). The mechanisms of insulin or IGF binding to their cognate receptors were originally proposed on the basis of extensive mutagenesis studies only (47, 48). More recently, however, several reports based on the crystal structures of the insulin·IR complexes (49, 50), “activated” insulin analogs (51–53), and the first bound structure of IGF-I through complexation with a IR/IGF-1R hybrid construct (54) have revealed the binding mode of the hormones at the receptor site 1 represented by the L1 subunit and α-CT segment. However, details of the precise arrangement of the C-domain of bound IGF-I are currently unknown, but structural rearrangement of this region in conjunction with the α-CT region has been proposed to be necessary to prevent unfavorable steric clashes. Moreover, the C-domain is a region with major differences between IGFs, both in the amino acid composition and length (Fig. 1), probably being a key determinant of receptor binding specificity.

Both insulin and IGF-I have been extensively studied through the preparation and functional analysis of numerous analogs (for extensive reviews, see Refs. 46, 48, and 55), whereas the structure-function of IGF-II is less developed (15, 56–62). To gain greater insight into the structural basis of IGF-II binding specificity to IR-A and IGF-1R, we generated a series of mutants containing amino acid substitutions within the B- and C-domains of IGF-II. These were designed to make IGF-II more IGF-I-like (Fig. 1) and were tested through binding affinities to their cognate receptors. This was enabled by the development of a new, efficient, and cost-effective protocol for recombinant production of IGF-II analogs in sufficient quantities for structural characterization by NMR. Our data revealed that the newly prepared IGF-II analogs display conserved or slightly increased IGF-1R affinities but markedly reduced IR-A affinities, which correlates with the specific conformational changes in the structurally elusive C-domain of IGF-II.

## Results

### 

#### 

##### Recombinant Production of IGF-II

A prerequisite for this study was the efficient production of correctly folded IGF-II, which would serve as a reference molecule as well as a platform for the design and production of new IGF-II analogs. This was achieved by recombinant IGF-II expression in *Escherichia coli* as a fusion with an N-terminal and cleavable His_6_-tagged GB1 protein (immunoglobulin binding domain B1 of streptococcal Protein-G) (63, 64). This technique provided high yields (0.8–1.8 mg liter^−1^ of culture) of IGF-II analogs with only a single additional glycine residue cloning artifact at the N terminus. The fusion protein was successfully expressed in *E. coli* and purified using immobilized metal ion affinity chromatography (supplemental Fig. S1). Two major peaks were observed; the first eluted at lower concentrations of imidazole (110–160 mm; fractions 1–2 in supplemental Fig. S1) and consisted of folded and misfolded monomeric IGF-II with slightly different migration of two bands observable in non-reducing SDS-PAGE (supplemental Fig. S1). The second peak eluted at higher concentrations of imidazole and consisted of multimeric forms (310–480 mm; fractions 4–5 in supplemental Fig. S1). Both monomeric and multimeric fusion proteins were subsequently cleaved using TEV protease under redox conditions of oxidized and reduced glutathione. Interestingly, the moderate reducing environment triggered disulfide bond reshuffling that resulted in liberation of monomeric IGF-II from multimeric aggregates. Following cleavage, IGF-II was separated from the His_6_-tagged GB1 and TEV by immobilized metal ion affinity chromatography. RP-HPLC of this crude IGF-II product consisted of one major peak and two to four minor peaks (supplemental Fig. S1). The retention time of the major protein peak was nearly identical to that observed for native human IGF-II, and the correct molecular weight of the recombinantly produced purified IGF-II protein with formed disulfide bonds was confirmed by high resolution mass spectrometry. Both forms, monomeric and multimeric, yielded the desired product of correct mass and were combined after the correct protein fold was confirmed by 1D ^1^H NMR (supplemental Fig. S2) and ^1^H-^15^N HSQC that was highly similar to the previously published data (65).

In total, six IGF-II analogs were designed to determine the effects of IGF-I motif incorporation into IGF-II. The modifications were as follows: (i) a point mutation at position Ser^29^ for Asn ([N29]IGF-II), (ii) an insertion of Gly-Ser after Arg^34^ ([R34_GS]IGF-II), (iii) an insertion of Pro-Gln after Ser^39^ ([S39_PQ]IGF-II), (iv) a combination of both insertions ([R34_GS,S39_PQ]IGF-II), (v) a combination of S29N mutation with Pro-Gln insertion ([N29,S39_PQ]IGF-II), and (vi) a combination of S29N mutation with both insertions ([N29,R34_GS,S39_PQ]IGF-II). All analogs gave comparable RP-HPLC elution profiles (data not shown) with that of IGF-II (supplemental Fig. S1) with one major product and several minor peaks. The characterization of minor by-products was prevented by their relatively low yields.

The structural integrity of the six analogs was confirmed using ^1^H NMR and far-UV circular dichroism as illustrated in supplemental Figs. S2 and S3. The CD spectra obtained for prepared analogs are similar to the broadly α-helical secondary structure profile obtained for non-modified IGF-II. The presence of the expected tertiary structure was further confirmed by 1D ^1^H (supplemental Fig. S2) NMR spectra, and each analog compared well with the native IGF-II profile.

##### Receptor Binding

**FIGURE 2.**
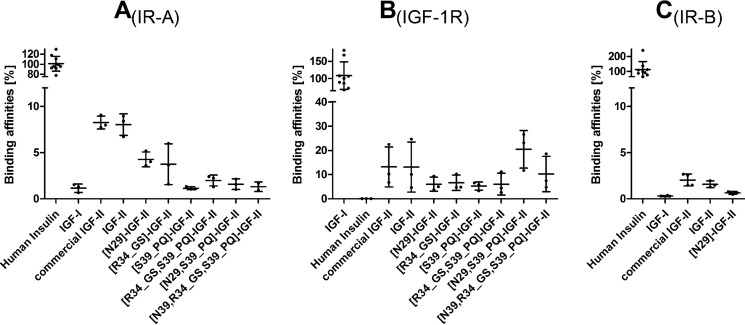
**Summary of receptor binding affinities.** Shown is a bar plot representation of relative binding affinities (from Table 1) of native hormones and IGF-II analogs prepared in this work for human IR-A (*A*), IGF-1R (*B*), and IR-B (*C*). *Error bars* represent S.D.

The binding affinities of the IGF-II analogs toward human IR-A and IGF-1R together with binding affinities of selected analogs to IR-B are summarized in Table 1 and Fig. 2. The corresponding binding curves are shown in supplemental Figs. S4–S6.

**TABLE 1:** **The receptor binding affinities of hormones and IGF-II analogs reported in this work** The values of *K_d_* and relative binding affinities of human insulin, IGF-I, IGF-II, and IGF-II analogs were determined for human IR-A in membranes of human IM-9 lymphocytes and for human IR-B and human IGF-1R in membranes of mouse fibroblasts. Relative receptor binding affinity is defined as (*K_d_* of human insulin or IGF/*K_d_* of analog) × 100. ND is not determined.

Analog	*K_d_* ± S.E. (nm) (*n*) for human IR-A in IM-9 lymphocytes	Relative binding affinity for human IR-A	*K_d_* ± S.E. (nm) (*n*) for human IGF-1R in mouse fibroblasts	Relative binding affinity for human IGF-1R	*K_d_* ± S.E. (nm) (*n*) for human IR-B in mouse fibroblasts	Relative binding affinity for human IR-B
		%		%		%
Commercial human insulin	0.43 ± 0.02 (5)	100 ± 5	292 ± 31 (3)*^a^*	0.08 ± 0.01	0.67 ± 0.17 (5)*^b^*	100 ± 18
	0.24 ± 0.02 (5)	100 ± 8			0.67 ± 0.08 (4)*^a^*	100 ± 12
Commercial human IGF-I	23.8 ± 6.6 (3)*^b^*	1.0 ± 0.3*^c^*	0.24 ± 0.05 (5)*^a^*	100 ± 21	224 ± 16 (4)*^b^*	0.3 ± 0.0 *^d^*
			0.25 ± 0.01 (4)	100 ± 4		
Commercial human IGF-II	2.92 ± 0.14 (3)*^b^*	8.2 ± 0.4*^c^*	2.32 ± 0.72 (3)*^b^*	10.8 ± 3.3*^e^*	35.5 ± 5.6 (4)*^b^*	1.9 ± 0.3*^d^*
IGF-II	3.03 ± 0.27 (3)	7.9 ± 0.7*^c^*	2.29 ± 1.04 (4)	10.9 ± 5.0*^e^*	43.7 ± 5.3 (3)	1.5 ± 0.2*^d^*
[N29]IGF-II	10.3 ± 1.1 (3)	4.2 ± 0.4*^f^*	4.57 ± 1.09 (3)	5.3 ± 1.3*^g^*	108 ± 16 (3)	0.6 ± 0.1*^h^*
[R34_GS]IGF-II	15.4 ± 6.0 (3)	2.8 ± 1.1*^f^*	4.13 ± 0.90 (3)	5.8 ± 1.3*^g^*	ND	ND
[S39_PQ]IGF-II	38.0 ± 2.9 (3)	1.1 ± 0.1*^f^*	5.00 ± 1.10 (3)	4.8 ± 1.1*^g^*	ND	ND
[R34_GS,S39_PQ]IGF-II	23.4 ± 4.8 (3)	1.8 ± 0.4*^f^*	5.68 ± 2.13 (3)	4.2 ± 1.6*^g^*	ND	ND
[N29,S39_PQ]IGF-II	16.8 ± 3.8 (3)	1.4 ± 0.3*^c^*	1.33 ± 0.36 (3)	18.8 ± 5.1*^e^*	ND	ND
[N29,R34_GS,S39_PQ]IGF-II	19.9 ± 5.5 (2)*^i^*	1.2 ± 0.3*^c^*	3.19 ± 1.08 (3)	7.8 ± 2.7*^e^*	ND	ND

*^a^* From Vikova *et al.* (87).

*^b^* From Krizkova *et al.* (2).

*^c^* Relative to human insulin *K_d_* value of 0.24 ± 0.02 (*n* = 5).

*^d^* Relative to human insulin *K_d_* value of 0.67 ± 0.12 (*n* = 5).

*^e^* Relative to human IGF-I *K_d_* value of 0.25 ± 0.01 (*n* = 4).

*^f^* Relative to human insulin *K_d_* value of 0.43 ± 0.02 (*n* = 5).

*^g^* Relative to human IGF-I *K_d_* value of 0.24 ± 0.05 (*n* = 5).

*^h^* Relative to human insulin *K_d_* value of 0.67 ± 0.08 (*n* = 4).

*^i^* This *K_d_* value represents mean of two independent measurements ±range.

##### IR-A Binding Affinities

All modifications led to a significantly impaired IR-A binding, ranging from 4.2 to 1.1% of the insulin affinity when compared with IGF-II (7.9%). The [N29]IGF-II B-domain mutant gave a 2-fold reduction in IR-A affinity, whereas the analogs with C-domain insertions exhibited stronger negative effects. [R34_GS]IGF-II showed an almost 3-fold reduction in binding (2.8%), whereas [S39_PQ]IGF-II showed an 8-fold reduction. All of the analogs bearing the Pro-Gln motif were significantly less active (1.1–1.8%), and further combinations did not appear to have any additive effect.

##### IGF-1R Binding Affinities

An insertion of IGF-I-like features, S29N, Gly-Ser, Pro-Gln alone, or a combination of Gly-Ser and Pro-Gln, within the IGF-II molecule led rather unexpectedly to a moderate decrease of binding potency toward IGF-1R (Table 1 and Fig. 2). However, the Pro-Gln insertion combined with the S29N mutation resulted in an increase in binding potency to that of 18.8% to IGF-I in comparison with IGF-II (10.9%). In contrast, this effect was negated when the S29N mutation was combined with both insertions.

##### IR-B Binding Affinities

Both reference molecules, commercial IGF-II and our recombinant IGF-II, show similar binding potency for IR-B compared with IGF-I (1.9 and 1.5% of human insulin, respectively; ∼40 nm; Table 1). The IR-B binding affinity of [N29]IGF-II dropped to almost one-third of the potency obtained for IGF-II (0.6%; 108 nm).

##### Structural Characterization of IGF-II Analogs by NMR Spectroscopy

We selected two IGF-II analogs with the most pronounced impact on receptor binding [S39_PQ]IGF-II (with lowest IR-A and IGF-1R binding) and [N29,S39_PQ]IGF-II (with decreased IR-A and enhanced IGF-1R binding) (Table 1 and Fig. 2) for NMR structural characterization to understand the molecular basis of Pro-Gln and S29N modifications.

Undesirable dynamic and aggregation behavior of IGF-II severely affects the quality of NMR spectra of this protein and would prevent the accurate structural determination required for a detailed comparison between these analogs. Previously, it has been shown that upon binding to an engineered high affinity Domain 11 (D11) of the IGF-2R the spectral properties of IGF-II improve dramatically (65). The fact that the IGF-II modifications reported here are distributed on the opposing face to the D11 binding site allowed this system to be utilized for structural studies of the B- and C-domains. As expected, the binding of either ^15^N- or ^13^C/^15^N-labeled IGF-II analogs to unlabeled D11 led to a significant line narrowing of the NMR signals as illustrated in supplemental Fig. S7 despite the more than a 2-fold increase in the total molecular mass of the system. First, we determined the structure of the D11-bound unmodified IGF-II that was utilized in the structural analysis of IGF-II analogs. As expected, it is highly similar to the previously published structure (65) with some regions being more resolved, especially around the sites modified in the analogs, reflecting the substantially higher number of experimental restraints (1039 *versus* 764 unambiguous NOE restraints (supplemental Table S1 and Ref. 65)). Next, we verified that binding to D11 did not significantly affect the IGF-II C-domain and C-terminal portion of the B-domain by comparison of assigned 2D ^1^H-^15^N HSQC spectra of free and D11-bound [S39_PQ]IGF-II (supplemental Fig. S8). Although significant chemical shift perturbations were observed over the A-domain and the first 75% of the B-domain, the regions containing the mutations showed very small or negligible chemical shift perturbations.

**FIGURE 3.**
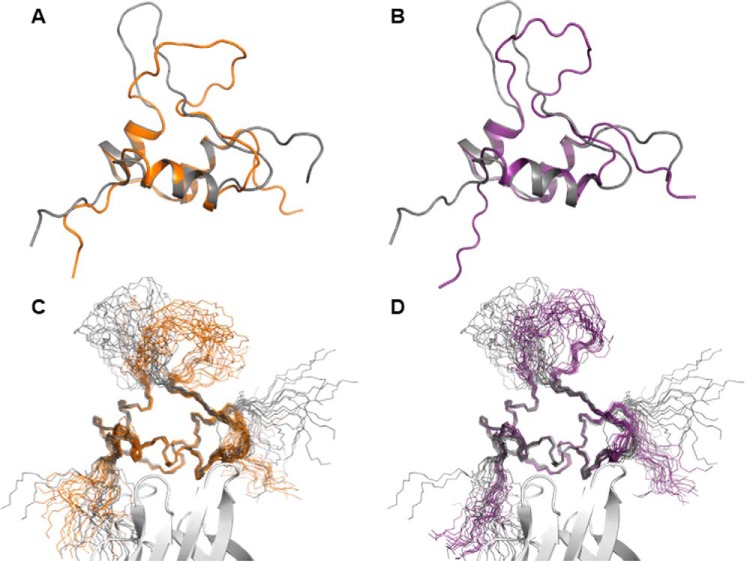
**Solution structures of [S39_PQ]IGF-II (*orange*) and [N29,S39_PQ]IGF-II (purple) compared with non-modified IGF-II (gray).** *A* and *B* show representative structures of the Domain 11-bound IGF-II analogs, and *C* and *D* show sets of 20 converged structures bound to D11 (*white*). The insertion of Pro-Gln in the C-domain after position 39 led to a significant structural rearrangement of the semiflexible loop.

**FIGURE 4.**
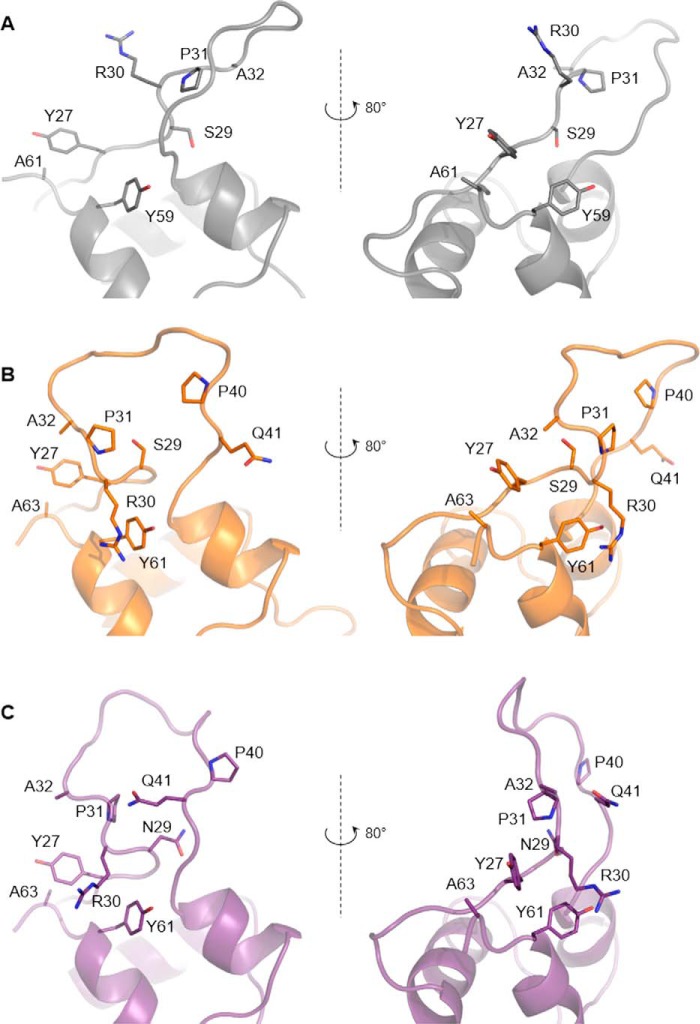
**Structural impact of the IGF-II modifications.** Non-modified IGF-II (*A*; *gray*) is compared with [S39_PQ]IGF-II (*B*; *orange*) and [N29,S39_PQ]IGF-II (*C*; *purple*), revealing different spatial orientation of highlighted residues. In particular, the rearrangement of the C-domain is driven by repositioning of Ala^32^ toward Tyr^27^ and Arg^30^ toward Tyr^61^ (Tyr^59^ in non-modified IGF-II) supported by additional contacts within this area.

**FIGURE 5.**
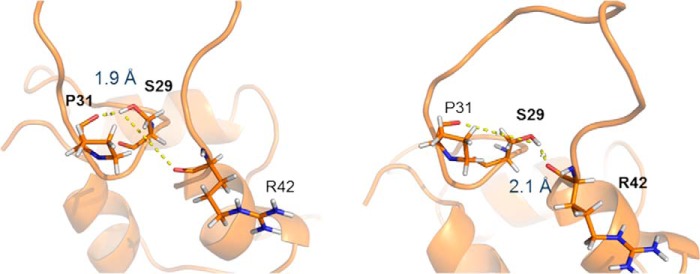
**The formation of stabilizing hydrogen bond in [S39_PQ]IGF-II.** The hydroxyl proton from the Ser^29^ side chain is stabilizing the C-loop via a hydrogen bond to the backbone carboxyl groups either from Pro^31^ or Arg^42^.

Both analogs, [S39_PQ]IGF-II and [N29,S39_PQ]IGF-II, preserved their overall structural organization with the three highly conserved α-helices further stabilized by three disulfide bonds. As expected, the D11 binding interface on the IGF-II analogs was not perturbed by the modifications, and structural changes were restricted to the modification sites (Fig. 3). In both analogs, the C-domain insertion led to a significant change in the conformational space sampled by this region of the protein compared with unmodified IGF-II with the main differences residing between residues 29 and 42. Detailed analysis (Fig. 4) revealed that the insertion of Pro-Gln after Ser^39^ led to increased conformational freedom within the C-loops of both analogs that generated a rearrangement stabilized by several new packing interactions in the remote part of the C-domain. In the native IGF-II sequence, Tyr^27^ points away from the C-loop and forms hydrophobic contacts with Ala^61^, whereas the C-loop is unrestrained by additional contacts to the other parts of IGF-II (Fig. 4*A*). By contrast, the aromatic ring of Tyr^27^ forms contacts to the methyl group of Ala^32^ in [S39_PQ]IGF-II (Fig. 4*B*) and Arg^30^ and Pro^31^ in [N29,S39_PQ]IGF-II (Fig. 4*C*). Arg^30^ is no longer unrestrained in these analogs and interacts with the aromatic ring of Tyr^61^ (Tyr^59^ in unmodified IGF-II) via a cation-π interaction. These new hydrophobic contacts lead to the formation of a better defined C-loop that bends around the bulky side chains of Tyr^27^ and Tyr^61^ of both C-domain-modified analogs (Fig. 4, *B* and *C*). In comparison with unmodified IGF-II, the extended C-domain in both analogs is spatially constrained and bent toward the triad of aromatic residues at the C terminus of the B-domain (Phe^26^, Tyr^27^, and Phe^28^). Ser^29^ in IGF-II (Fig. 4*A*) is located at the hinge of the semiflexible loop with no significant contacts to neighboring residues. The Pro-Gln extension in [S39_PQ]IGF-II led to the repositioning of Ser^29^ in close proximity to Tyr^27^, although there are no observed NOE contacts between Ser^29^ protons and Tyr^27^ or surrounding residues. However, the hydroxyl proton from its side chain may be involved in hydrogen bonds, *e.g.* with the backbone carboxyl groups either from Pro^31^ (<2.8 Å in half of the structures), which is closer in the extended loop, or from Arg^42^ (<2.8 Å in a quarter of the structures) at the opposite side of the loop (Figs. 4*B* and 5). The modification of Ser^29^ to Asn^29^ in [N29,S39_PQ]IGF-II led to a loss of this hydrogen bond and a subtle conformational rearrangement of the C-loop backbone. In addition, the Asn^29^ side chain is pointing out of the C-loop and is fully solvent-exposed with NOE contacts between the NH_2_ group from the Asn^29^ side chain and H^β2^ from Phe^28^, perhaps further stabilizing the cluster of contacts between the C-domain and aromatic triad that in turn might stabilize the additional interactions seen between Tyr^27^ and Arg^30^/Pro^31^ (Fig. 4*C*) that were not observed for the [S39_PQ]IGF-II analog.

## Discussion

IGF-II is capable of binding to both IR-A and IGF-1R with single digit nanomolar affinity (*K_d_* ∼ 3 nm; Table 1) and to IR-B with lower affinity (∼40 nm; Table 1). Although the binding affinities of the “parent” ligands, insulin and IGF-I, toward their cognate receptors are in the subnanomolar range (Table 1), IGF-II can still effectively signal through both IR-A and IGF-1R receptors or their hybrid forms *in vivo* (66, 67), which may trigger unfavorable biological responses. The knowledge about structural elements within these hormones responsible for differential binding specificity to each receptor could open a new path to the development of receptor-selective IGF and insulin analogs with potential medical applications. The analogs prepared and structurally characterized in this work were designed to investigate the effects of introducing unique IGF-I motifs (*i.e.* Asn^26^, Gly^30^-Ser^31^, and Pro^35^-Gln^36^; Fig. 1) to IGF-II on receptor binding behavior. We hypothesized that such modifications may negatively affect the hormone's binding potency toward IR-A while enhancing the binding affinity for IGF-1R. Moreover, there are no reported analogs with the mutation of Ser^29^ in IGF-II, and there are only a few studies regarding alterations in the C-domain (57, 59).

The development of an efficient protocol for IGF-II production was a key step in being able to reliably prepare the IGF-II analogs. The total chemical synthesis of IGF-II is extremely difficult and time-consuming due to the length and unfavorable composition of the IGF-II sequence (68). The most frequently used recombinant approach, analogous to the production of IGF-I (69, 70), is based on preparation of a fusion comprising porcine growth hormone N-terminal residues 1–11 (plus N-terminal Met), a subtilisin-specific cleavage sequence (Val-Asn-Phe-Ala-His-Tyr↓), and human IGF-II (71). However, specifically mutated subtilisin (H64A) used for the procedure is no longer commercially available. We therefore chose an alternative approach that includes an “on-column” refolding step of denatured IGF-II in a fusion with His_6_-tagged GB1 protein (63, 64). Subsequent cleavage of the fusion protein in a redox environment and RP-HPLC purification yields IGF-II with only a single additional glycine residue at the N terminus. This improves on the recently reported recombinant method that leaves three surplus N-terminal amino acids (glycine, alanine, and methionine) (65, 72) and therefore reduces uncertainty in interpreting structure and function of this protein in biological assays.

The binding affinities of recombinantly produced IGF-II toward IR-A, IR-B, and IGF-1R correlate with the values obtained for commercial IGF-II (Table 1 and Fig. 2). These comparable binding characteristics confirmed the correct disulfide pairing as misfolded IGFs do not bind to IGF-1R or IR-A with a measurable affinity (27, 73, 74). This method therefore leads to a rapid and cost-effective preparation of authentic IGF-II, providing us and others with an essential tool for studying IGF-II-related structure and function.

Our initial goal to reduce IR-A affinity of IGF-II was successful as all six IGF-II analogs showed reduced IR-A binding (Table 1) with four of these showing low affinity similar to IGF-I. The most significant was [S39_PQ]IGF-II with an ∼7–8-fold reduction in affinity compared with IGF-II. Interestingly, this reduction was greater than the effect of swapping the entire IGF-II C-domain for IGF-I C-domain (3.7-fold) (57). Our data and data of others (57, 59) suggest that there are two main factors affecting the binding potency of IGF-II to IR-A. First, a longer C-domain may introduce structural restrictions during binding to the insulin receptor. This is in agreement with the finding that IGF-I analogs with a shorter C-domain exhibit enhanced binding affinity to IR-A (75). The second factor relates to specific C-domain amino acids (*e.g.* Pro^39^ in IGF-I), which may affect the structure of the C-domain main chain and therefore binding to IR-A.

Although we only tested a single analog for binding to IR-B, the 2-fold reduction in binding observed for [N29]IGF-II suggests a similar sensitivity to changes in the C-domain (Table 1). Hence, we have not further pursued testing IR-B affinities of analogs and we focused on binding to IGF-1R.

The combination of the Pro-Gln insertion with S29N mutation in [N29,S39_PQ]IGF-II (Table 1 and Fig. 2) led to an analog exhibiting higher binding affinity to IGF-1R compared with native IGF-II. Our data suggest that the IGF-II specificity toward IGF-1R is determined by the amino acid composition of the C-domain rather than its length as demonstrated by the relatively lower binding affinity of the [R34_GS,S39_PQ]IGF-II analog. The selected mutations do not completely recover IGF-I-like binding to IGF-1R and cannot counterbalance the absence of other important IGF-I determinants (*e.g.* IGF-I Tyr^31^ (76–78)). Nonetheless, the almost doubling in IGF-1R binding affinity of [N29,S39_PQ]IGF-II analog together with its markedly lowered affinity for IR-A resulted in almost 10-fold enhanced IGF-1R/IR-A binding specificity in comparison with IGF-II.

The comparison of D11-bound structures of IGF-II, [S39_PQ]IGF-II and [N29,S39_PQ]IGF-II, revealed that both analogs differ from IGF-II in the orientation and structuring of their C-loops (Figs. 3 and 4). The significant and similar displacement of the C-loops in both [S39_PQ]IGF-II and [N29,S39_PQ]IGF-II together with their more open C-loop conformations can be attributed to the effect of their PQ inserts. Moreover, the C-loop loops back to generate a turn stabilized by contacts between Tyr^27^ and Ala^32^ and a hydrogen bond between Ser^29^ and Pro^31^ or Arg^42^ in [S39_PQ]IGF-II (Figs. 4*B* and 5). The absence of this hydrogen bond due to the S29N mutation in [N29,S39_PQ]IGF-II might be compensated for by Pro^31^ packing against Tyr^27^ (Fig. 4*C*). A comparable decrease in IR-A binding affinities of [S39_PQ]IGF-II and [N29,S39_PQ]IGF-II in comparison with IGF-II indicates it is caused mainly by their similarly altered C-loop structures rather than S29N mutation, which is well tolerated by IR-A.

**FIGURE 6.**
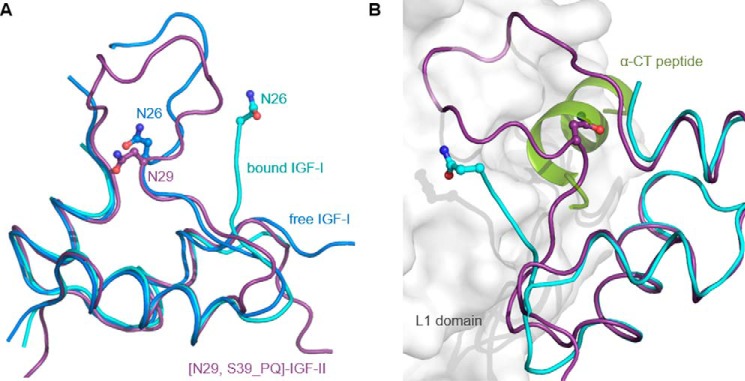
**A superposition of free or hybrid IR/IGF-1R fragment-bound forms of IGF-I with [N29,S39_PQ]IGF-II.** *A*, an overlay of the backbone of free human IGF-I (Protein Data Bank code 1GZR; in *blue*) with [N29,S39_PQ]IGF-II (in *purple*) and IGF-I from a complex with the L1 domain from IR and IGF-1R α-CT peptide (Protein Data Bank code 4XSS; in *cyan*). The positions of Asn^26^ in IGF-I and Asn^29^ in IGF-II side chains are highlighted. *B*, the crystal structure (Protein Data Bank code 4XSS) of IGF-I (in *cyan*) in a complex with IR L1 domain (in *white*) and IGF-1R α-CT peptide (in *green*) overlaid with [N29,S39_PQ]IGF-II in *purple*.

In the crystal structure of human IGF-I (Protein Data Bank code 1GZR) (29), the Asn^26^ side chain is solvent-exposed at the interface of the B- and C-domains with the Asn^26^ presenting a potential polar hot spot. An equivalent Asn^29^ in [N29,S39_PQ]IGF-II is in a similar position but is less exposed due to a partial overlap by the rearranged C-loop (Fig. 6*A*). Asn^26^ is at the C terminus of the IGF-I B-domain, which is structurally altered upon binding to IGF-1R or IR (54) (Fig. 6, IGF-I receptor-bound structures in *cyan*). Analogous structural events are observed upon insulin binding to IR (50, 53), and it can be expected that receptor-driven activation of IGF-II is similar. In the Menting *et al.* (54) structure (Protein Data Bank code 4XSS), Asn^26^ is the last IGF-I B-domain residue resolved in the complex with the hybrid IGF-IR/IR where it has been captured in the binding site formed from the IGF-IR α-CT and IR L1 domains (Fig. 6*B*). However, the structure of the complex did not reveal any specific contacts between IGF-I Asn^26^ and IR L1 domain or IGF-IR α-CT. However, it cannot be ruled out that Ser^29^ within the IGF-II molecule (or Asn^29^ in [N29,S39_PQ]IGF-II) may be involved in direct contacts to IGF-1R, and this hypothesis could be supported by a positive effect of S29N mutation on IGF-1R binding affinity of [N29,S39_PQ]IGF-II. Hence, Ser^29^ may represent an important site for engineering of the IGF-1R binding specificity in IGF-II analogs.

## Concluding Remarks

We have developed a straightforward protocol for the production of recombinant IGF-II with an additional glycine at the N terminus. We prepared six IGF-II analogs with IGF-I-like mutations. All these mutants have markedly reduced affinity for IR-A, especially those analogs with Pro-Gln insertions in the C-domain. Moreover, one of the analogs, [N29,S39_PQ]IGF-II, shows the enhanced binding affinity for IGF-1R in comparison with IGF-II due to the synergistic effect of Pro-Gln insertion and S29N point mutation. Consequently, this analog has almost 10-fold enhanced IGF-1R/IR-A binding selectivity in comparison with IGF-II. Structural characterization of selected analogs revealed that the conformational rearrangement of the C-loop induced by insertion of two residues from IGF-I is manifested in the reduced affinity for IR-A. A combination of the effect of this insertion with an additional IGF-I like substitution, S29N, driving the additional subtle rearrangement of the C-loop forms a structural basis for the increased binding affinity of [N29,S39_PQ]IGF-II for IGF-1R. To our knowledge, the research reported here is a unique example of the determination of 3D structures of IGF-II analogs with modifications that have an impact on receptor binding affinities. Identification of structural determinants in IGFs and insulin that are responsible for specific binding to their cognate receptors is important for designing new, more specific hormone analogs with potential therapeutic applications.

## Experimental Procedures

### Recombinant Expression of IGF-II Analogs

The human IGF-II sequence was cloned into a modified pRSFDuet-1 expression vector fused with an N-terminal His_6_ tag, GB1 protein, and TEV protease cleavage site (Glu-Asn-Leu-Tyr-Phe-Gln↓Gly). An additional N-terminal Gly (−1) was incorporated to facilitate TEV cleavage. Mutation S29N, Gly-Ser insertion following Arg^34^, Pro-Gln insertion following Ser^39^, and a combination of both insertions were obtained by site-directed mutagenesis (QuikChange kit, Agilent Technologies) performed with appropriate mutagenic primers of the IGF-II sequence subcloned into the pBluescript vector. After sequence verification, the mutant fragments were reintroduced into the full-length IGF-II in the expression vector. Constructs were transformed into *E. coli* BL21(λDE3) and cultivated using LB medium or minimal medium containing [^15^N]ammonium sulfate and d-[^13^C]glucose. The bacterial culture was grown at 37 °C to an optical density (550 nm) of ∼1, induced with 1 mm isopropyl β-d-1-thiogalactopyranoside, and further cultured for 4–5 h. Cells were harvested by centrifugation for 20 min at 4,000 × *g*, and cell pellets were stored at −20 °C prior to further processing.

### Isolation of Inclusion Bodies

Cells pellets were resuspended in lysis buffer (50 mm Tris-HCl, pH 8.0, 50 mm NaCl, 5 mm EDTA, 50 μm PMSF) using 10 ml of buffer/1 g of biomass and homogenized by three passes through an Avestin EmulsiFlex-C3® apparatus at 4 °C and homogenization pressure of 1,200 megapascals. Inclusion bodies from the cell lysate were obtained by centrifugation at 20,000 × *g* at 4 °C for 20 min and further washed as a suspension in a wash buffer (50 mm Tris-HCl, pH 8.0, 50 mm NaCl, 5 mm EDTA) with 0.1% (v/v) Triton X-100, sonicated in an ice bath, and centrifuged (20,000 × *g*, 4 °C, 20 min). The wash procedure was repeated in the absence of 0.1% (v/v) Triton X-100, and wet paste consisting of inclusion bodies was stored at −20 °C.

### Purification of IGF-II and Analogs

The inclusion bodies were resuspended in a minimum volume (2 ml/g of wet paste) of 50 mm Tris-HCl, pH 8.0 buffer with 300 mm NaCl and sufficient β-mercaptoethanol to yield a final concentration of 0.02% (v/v) after the following dilution step. The suspension was gently diluted into 50 mm Tris-HCl, pH 8.0 buffer with 300 mm NaCl and 8 m urea to a final concentration of ∼1 g (wet weight of inclusion bodies)/50 ml and incubated for 2–3 h at room temperature with moderate stirring. The solution of the denatured fusion protein was then loaded onto an equilibrated HisTrap HP (5 ml) column connected to an ÄKTA FPLC® system (GE Healthcare), and after washing with 50 mm Tris-HCl, pH 8.0 buffer with 300 mm NaCl, the retained protein was eluted using a 0–500 mm imidazole gradient in 50 mm Tris-HCl, pH 8.0 buffer with 300 mm NaCl within 10 column volumes. The presence of the fusion protein in collected fractions was verified by SDS-PAGE and anti-His_6_ Western blotting, and the pooled fractions were dialyzed at 6 °C against 50 mm Tris-HCl, pH 8.0, 300 mm NaCl. The fusion partner was subsequently cleaved by an overnight TEV digestion in the presence of reduced and oxidized glutathione (1.5 mm GSH and 0.15 mm GSSG) at room temperature. Cleaved IGF-II was separated from the fusion protein by a gravity flow nickel chelating chromatography (HIS-Select Nickel Affinity Gel, Sigma-Aldrich) and further desalted on a Chromabond C_4_ column (Macherey-Nagel) using 80% CH_3_CN (v/v) with 0.1% TFA (v/v) for elution. The collected protein fraction was lyophilized; resuspended in 7% (v/v) acetic acid, 27% (v/v) CH_3_CN, 0.03% TFA (v/v); and purified on a semipreparative RP-HPLC column (Vydac 214TP510-C4, 250 × 10 mm) using a CH_3_CN/H_2_O gradient supplemented with 0.1% TFA (v/v). The separated fractions were lyophilized, the purity of products was analyzed by analytical RP-HPLC, and the identity of the products was verified by high resolution electrospray ionization mass spectrometry (LTQ Orbitrap XL, Thermo Fisher Scientific, Waltham, MA).

### NMR Spectroscopy

All NMR data for free IGF-II and analogs were acquired at 25 °C using 600- and 850-MHz Bruker Avance II spectrometers, both of which were equipped with ^1^H/^13^C/^15^N cryoprobes. To confirm the correct fold of IGF-II analogs, 1D ^1^H spectra (unlabeled samples) and 2D ^1^H-^15^N HSQC spectra were acquired. The NMR spectra were collected using 350-μl samples of protein (75–380 μm) dissolved in 50 mm *d*_4_-acetic acid (pH 3.0), 5% D_2_O (v/v), 0.01% (w/v) NaN_3_. Data for IGF-II and analogs bound to a high affinity Domain 11 variant of IGF-2R (D11) (65, 72) were acquired from 350-μl samples of 200–400 μm IGF-II·D11 complex in acetate buffer (20 mm *d*_4_-acetic acid, pH 4.2, 5% D_2_O (v/v), 0.01% (w/v) NaN_3_) at 35 °C.

To determine the structure of either free or bound IGF-IIs, a series of double and triple resonance spectra (79, 80) were recorded on ^13^C/^15^N uniformly labeled IGF-II or analogs to determine essentially complete sequence-specific resonance backbone and side chain assignments. Constraints for ^1^H-^1^H distances were derived from 3D ^15^N-^1^H NOESY-HSQC and ^13^C-^1^H NOESY-HMQC, which were acquired using an NOE mixing time of 100 ms.

The family of converged structures was initially calculated using Cyana 2.1 (81). The combined automated NOE assignment and structure determination protocol was used to automatically assign the NOE cross-peaks identified in NOESY spectra and to produce preliminary structures. In addition, backbone torsion angle constraints, generated from assigned chemical shifts using the program TALOS+ (82), were included in the calculations. Subsequently, five cycles of simulated annealing combined with redundant dihedral angle constraints were used to produce sets of converged structures with no significant restraint violations (distance and van der Waals violations <0.2 Å and dihedral angle constraint violation <5°), which were further refined in explicit solvent using YASARA software with the YASARA force field (83). The structures with the lowest total energy were selected. Analysis of the family of structures obtained was carried out using the Protein Structure Validation Software suite (Northeast Structural Genomics consortium) and MOLMOL (84). The statistics for the resulting structures are summarized in supplemental Table S1.

### Circular Dichroism

CD spectra were measured in a quartz cuvette with an optical path length of 0.5 mm (Starna Cells) using a J-815 spectropolarimeter (Jasco, Japan) at room temperature. The far- and near-UV CD spectra were used to identify changes in protein secondary and tertiary structures. The spectral regions were 200–300 nm. The final spectra were obtained as an average of five accumulations. The spectra were corrected for the baseline by subtracting the spectra of the corresponding polypeptide-free solution. Analogs or IGF-II was dissolved and measured in 5% aqueous acetic acid (0.33 mg/ml; 45 μm).

### Receptor Binding Studies

Commercial human insulin and IGF-II were provided by Sigma-Aldrich, and human IGF-I was provided by Tercica.

### Human IM-9 Lymphocytes (Human IR-A Isoform)

Receptor binding studies with the insulin receptor in membranes of human IM-9 lymphocytes (containing only human IR-A isoform) were carried out, and *K_d_* values were determined according to the procedure described recently (85). Binding data were analyzed by Excel algorithms especially developed for the IM-9 cell system in the laboratory of Prof. Pierre De Meyts (developed by A. V. Groth and R. M. Shymko, Hagedorn Research Institute, Denmark; a kind gift of P. De Meyts) using a method of non-linear regression and a one-site fitting program and taking into account potential depletion of free ligand. Each binding curve was determined in duplicate, and the final dissociation constant (*K_d_*) of an analog was calculated from at least three (*n* ≥ 3) independently determined binding curves. The dissociation constant of human ^125^I-insulin was set to 0.3 nm.

### Mouse Embryonic Fibroblasts

#### 

##### Human IR-B Isoform

Receptor binding studies with the insulin receptor in membranes of mouse embryonic fibroblasts derived from IGF-I receptor knock-out mice that solely expressed the human IR-B isoform were performed as described in detail previously (86, 87). Binding data were analyzed, and the dissociation constant (*K_d_*) was determined with GraphPad Prism 5 software using a method of non-linear regression and a one-site fitting program and taking into account potential depletion of free ligand. *K_d_* values of analogs were determined and calculated by the same procedure as for IR-A.

##### Human IGF-1R

Receptor binding studies with the IGF-I receptor in membranes of mouse embryonic fibroblasts derived from IGF-1R knock-out mice and transfected with human IGF-1R were performed as described previously (86, 87). Binding data were analyzed, and the dissociation constants were determined and calculated by the same method as for IR-B. The dissociation constant of human ^125^I-IGF-I was set to 0.2 nm. Mouse embryonic fibroblasts expressing human IR-B or IGF-1R were a kind gift from Prof. Antonino Belfiore (University of Magna Grecia, Catanzaro, Italy) and Prof. Renato Baserga (Thomas Jefferson University, Philadelphia, PA). Here we should note that the use of bovine serum albumin (*e.g.* Sigma-Aldrich A6003) void of “IGF-binding-like” proteins, which interfere with these binding assays, is essential for the preparation of the binding buffer (88).

## Author Contributions

R. H. and K. Křížková contributed equally to the paper. R. H., K. Křížková, K. Kedrová, and I. S. carried out protein expression and purification. R. H., P. S., Z. T., and V. V. carried out NMR experiments and structure refinement. K. Křížková, K. Kedrová, M. C., and L. Ž. tested the analogs. M. F. and C. W. carried out DNA cloning. P. U. measured CD spectra. J. J. and L. Ž. conceived the study, designed experiments, and analyzed data. R. H., K. Křížková, M. P. C., J. J., V. V., and L.Ž. wrote the paper. All authors discussed the results and commented on the manuscript.

## Supplementary Material

Supplemental Data

## Footnotes

4 The abbreviations used are:

IGF: insulin-like growth factor
IR: insulin receptor
IR-A: insulin receptor isoform A
IR-B: insulin receptor isoform B
IGF-1R: insulin-like growth factor type I receptor
IGF-2R: insulin-like growth factor type II receptor
L1: leucine-rich repeat region
α-CT: C-terminal helix
GB1: immunoglobulin binding domain B1 of streptococcal Protein-G
TEV: tobacco etch virus
RP-HPLC: reversed phase HPLC
HSQC: heteronuclear single quantum coherence
D11: Domain 11
HMQC: heteronuclear multiple quantum coherence.
