# Supplementary material for: Probing Receptor Specificity by Sampling the Conformational Space of the Insulin-like Growth Factor II C-domain
Source: J Biol Chem. 2016 Aug 10;291(40):21234–45. doi: 10.1074/jbc.M116.741041 (PMC5076530; doi:10.1074/jbc.M116.741041)
Supplement: Supplemental Data [file supp_291_40_21234__index.html]

Probing Receptor Specificity by Sampling the Conformational Space of the Insulin-like Growth Factor II C-domain — Probing Receptor Specificity by Sampling the Conformational Space of the Insulin-like Growth Factor II C-domain — Receptor Specificity of IGF-II Analogs — Supplemental Data 

# Probing Receptor Specificity by Sampling the Conformational Space of the Insulin-like Growth Factor II C-domain

## Supplemental Data

- Supplementary Information (.pdf, 1.2 MB) - Revised supplementary Information
