# Supplementary material for: Probing Receptor Specificity by Sampling the Conformational Space of the Insulin-like Growth Factor II C-domain
Source: J Biol Chem. 2016 Aug 10;291(40):21234–45. doi: 10.1074/jbc.M116.741041 (PMC5076530; doi:10.1074/jbc.M116.741041)
Supplement: Supplemental Data [file 10.1074_M116.741041_jbc.M116.741041-1.pdf]

# Supplementary Information

for

# Probing receptor specificity by sampling the conformational space of the insulin-like growth factor II C-domain

Rozálie Hexnerová[‡§#], Květoslava Křížková[‡§#], Milan Fábry[‡$], Irena Sieglová[‡], Kateřina Kedrová[‡§], Michaela Collinsová[‡], Pavlína Ullrichová[†], Pavel Srb[‡], Christopher Williams[£], Matthew P. Crump[£], Zdeněk Tošner[§], Jiří Jiráček[‡], Václav Veverka[‡*] and Lenka Žáková[‡*]

[‡]Institute of Organic Chemistry and Biochemistry, Academy of Sciences of the Czech Republic, v.v.i., Flemingovo nám 2, 166 10 Prague 6, Czech Republic

[§]Faculty of Science, Charles University in Prague, Albertov 6, Prague, 128 43, Czech Republic

[†]Department of Analytical Chemistry, University of Chemistry and Technology, Prague, Technická 5, 166 28 Prague 6, Czech Republic

[$]Institute of Molecular Genetics, Academy of Sciences of the Czech Republic, v.v.i., Vídeňská 1083, 142 20 Prague 4, Czech Republic

[£]Department of Organic and Biological Chemistry, School of Chemistry, Cantock's Close, University of Bristol, Bristol BS8 1TS, United Kingdom

[#]Joint first authors

**Table of Contents**

Figure S1. . . . . . . . . . . . . . . . . . . . . . . . . . . . . . . . . . . . . . . . . . . . . . 2
Figure S2. . . . . . . . . . . . . . . . . . . . . . . . . . . . . . . . . . . . . . . . . . . . . . 3
Figure S3. . . . . . . . . . . . . . . . . . . . . . . . . . . . . . . . . . . . . . . . . . . . . . 4
Figure S4. . . . . . . . . . . . . . . . . . . . . . . . . . . . . . . . . . . . . . . . . . . . . . 5
Figure S5. . . . . . . . . . . . . . . . . . . . . . . . . . . . . . . . . . . . . . . . . . . . . . 6
Figure S6. . . . . . . . . . . . . . . . . . . . . . . . . . . . . . . . . . . . . . . . . . . . . . 6
Figure S7. . . . . . . . . . . . . . . . . . . . . . . . . . . . . . . . . . . . . . . . . . . . . . 7
Figure S8. . . . . . . . . . . . . . . . . . . . . . . . . . . . . . . . . . . . . . . . . . . . . . 8
Table S1. . . . . . . . . . . . . . . . . . . . . . . . . . . . . . . . . . . . . . . . . . . . . . 9

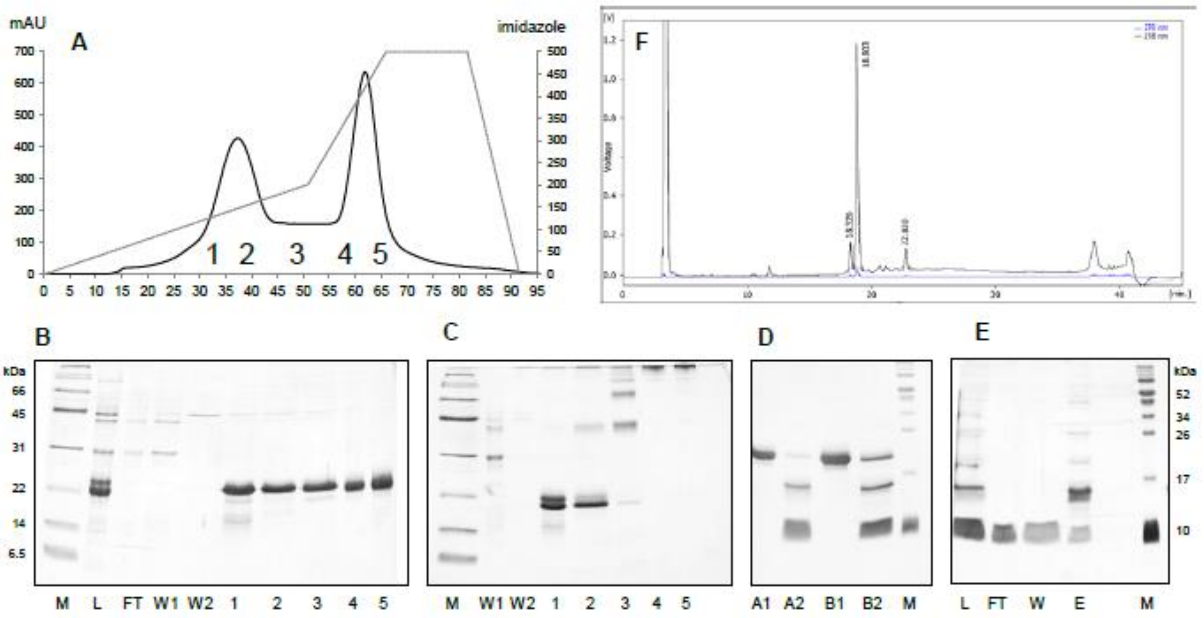


**Figure S1. Purification procedure for IGF-II analogs. A**. The elution profile from purification of denatured IGF-II in fusion with GB1 protein by IMAC. The material eluted in two major fractions (1-2 and 4-5) at two different imidazole concentrations. SDS-PAGE analysis of collected fractions (1-5) under reducing (**B**) and non-reducing (**C**) conditions revealing the presence of two monomeric isoforms (folded and misfolded) eluting at lower concentration of imidazole (150 mM) and multimeric aggregates eluting at higher imidazole concentration (400 mM). M, molecular weight standard; L, sample load; FT, flow through; W1 and W2, wash; 1-5, eluted fractions. Panel **D** shows reducing SDS-PAGE of the fusion partner cleavage by TEV protease. A1, monomeric fractions before TEV addition; A2, monomeric fractions after 24hrs of TEV digestion; B1, multimeric fraction before TEV addition; B2, multimeric fractions after 24hrs of TEV digestion; M, molecular weight standards. Panel **E** shows reducing SDS-PAGE of cleaved sample after nickel chelating chromatography. The cleaved IGF-II is present in FT and W fraction. L, sample load, FT, flow through; W, wash; E, elution; M, molecular weight standard. Panel **F** shows the final RP-HPLC purification of IGF-II separating forms with differently linked disulfide bonds.

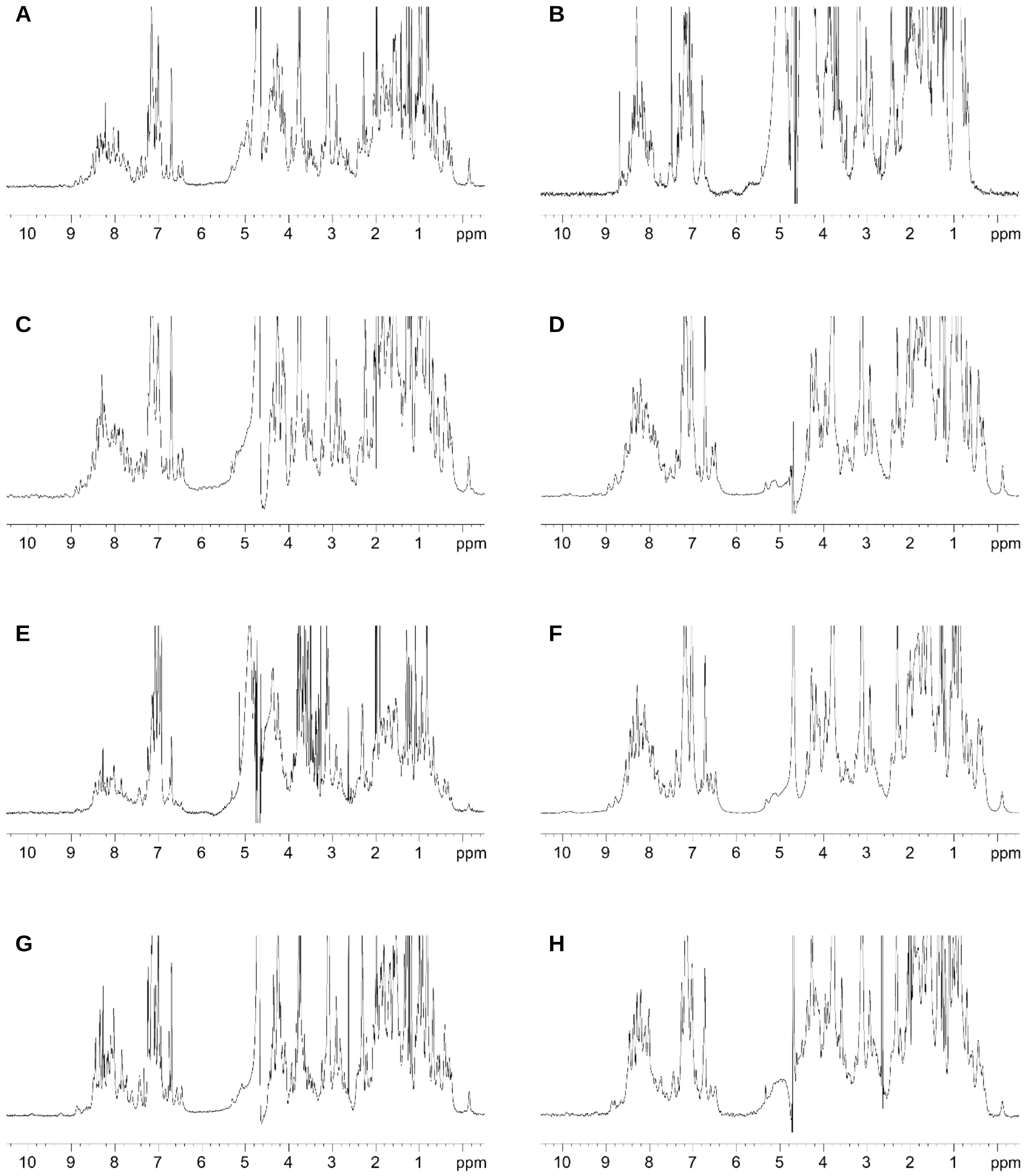


**Figure S2. ¹H NMR spectra of IGF-II analogues.** (**A**) IGF-II, (**B**) misfolded IGF-II, (**C**) [N29]-IGF-II, (**D**) [R34_GS]-IGF-II, (**E**) [S39_PQ]-IGF-II, (**F**) [R34_GS,S39_PQ]-IGF-II, (**G**) [N29, S39_PQ]-IGF-II, (**H**) [N29, R34_GS, S39_PQ]-IGF-II. The difference between correctly folded (A) and misfolded (B) IGF-II spectra was used for verification of correct protein folding of the IGF-II analogs (C-H). In particular, the presence of dispersed aromatic proton signals at 6.5 ppm and upfield shifted methyl signals between 0.5 and -0.2 ppm could be utilized to fingerprint correctly folded IGF-II.

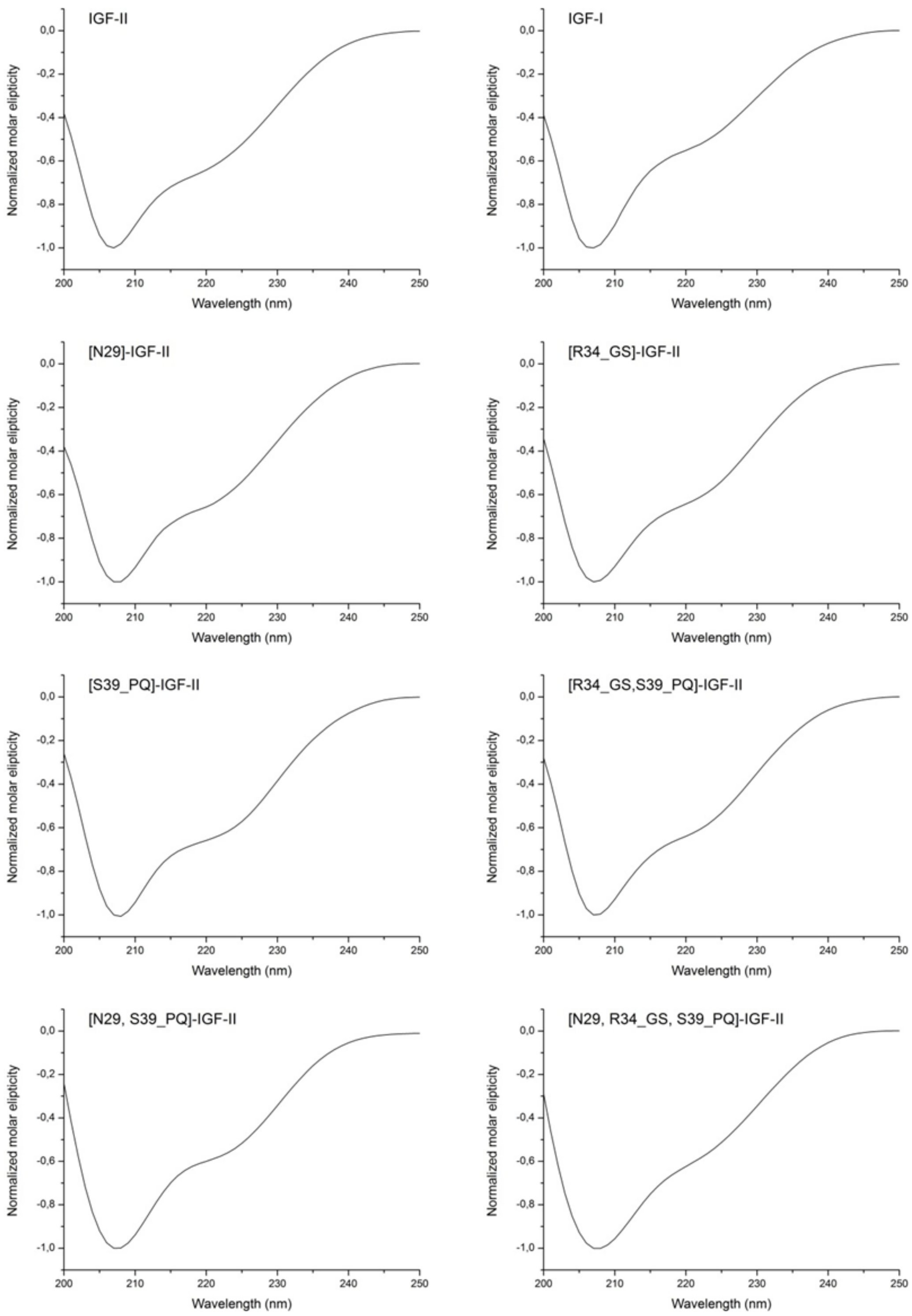


**Figure S3. Far UV circular dichroism spectra of IGF-I and studied IGF-II analogs normalized to 207 nm.** The curve profiles suggest highly similar presence of the α-helical secondary structure elements in the studied IGF-II analogs.

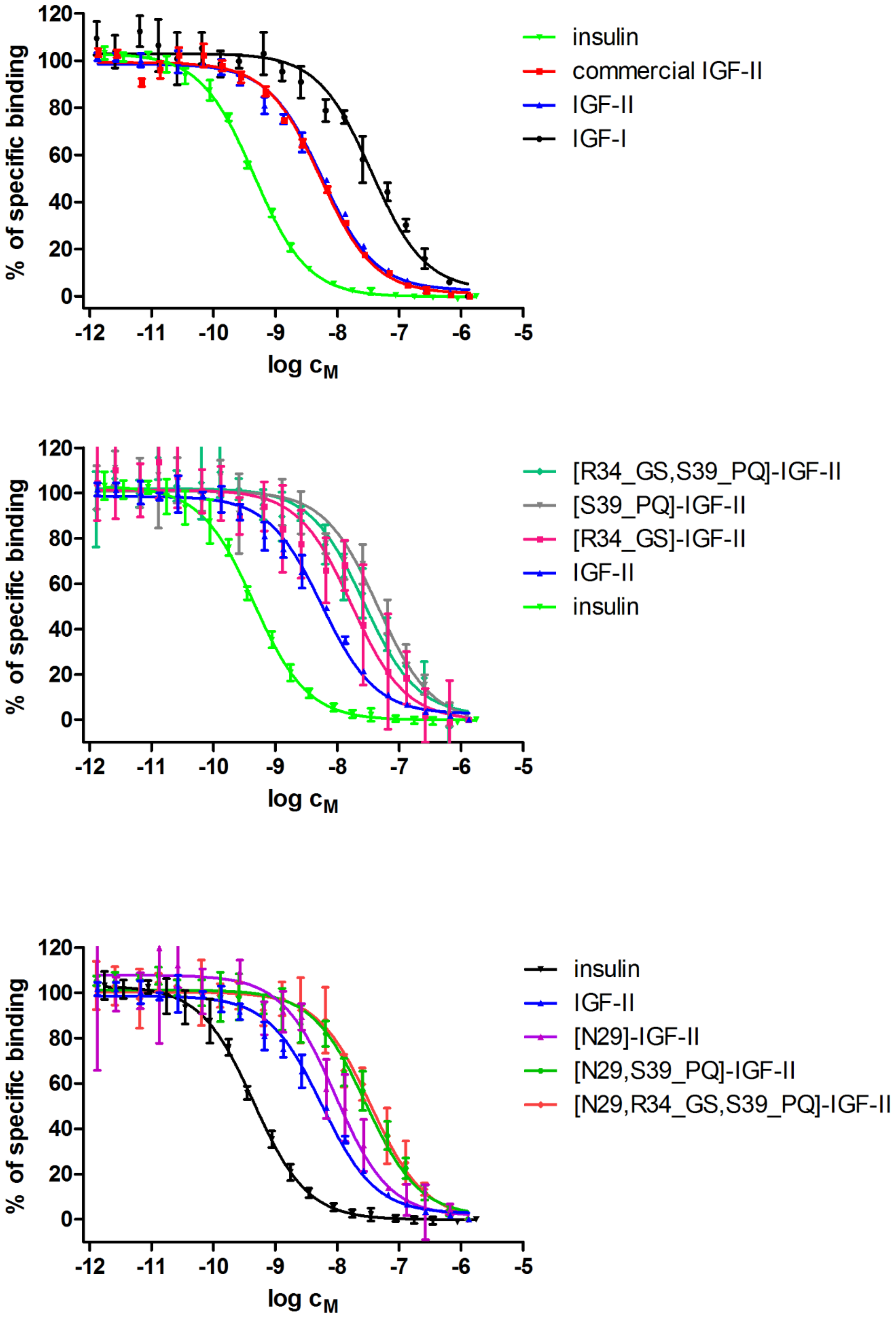


**Figure S4. Inhibition of binding of human [$^{125}$I]-insulin to IR-A in membranes of IM-9 cells by human insulin, IGF-I, IGF-II and IGF-II analogs.**

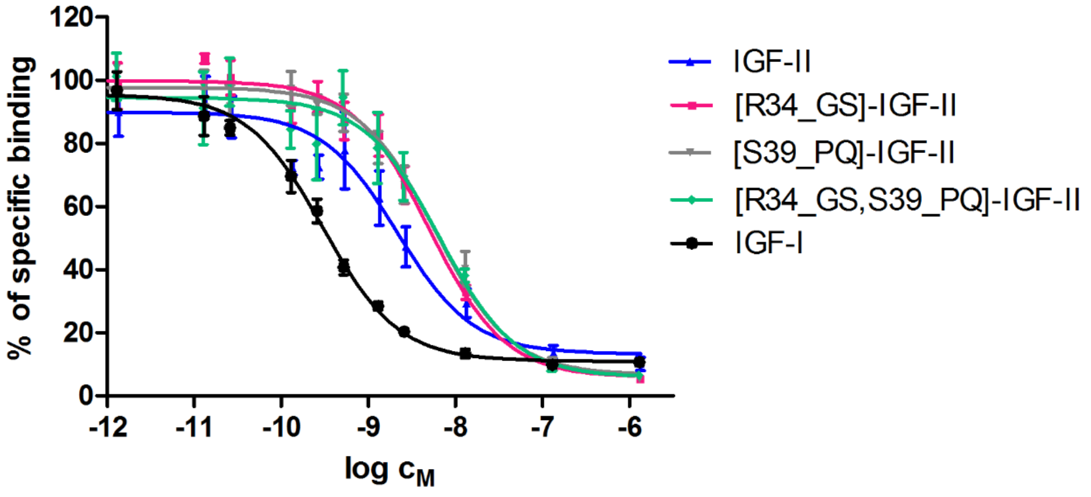


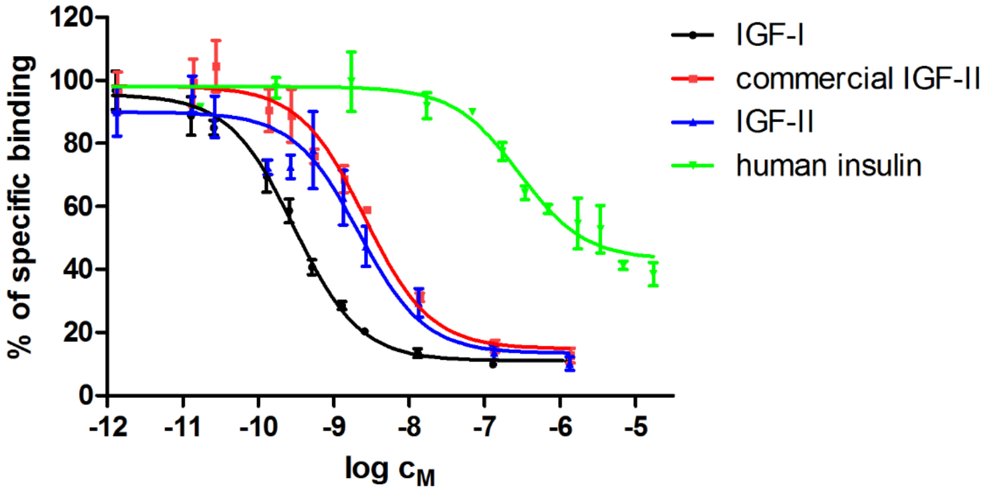


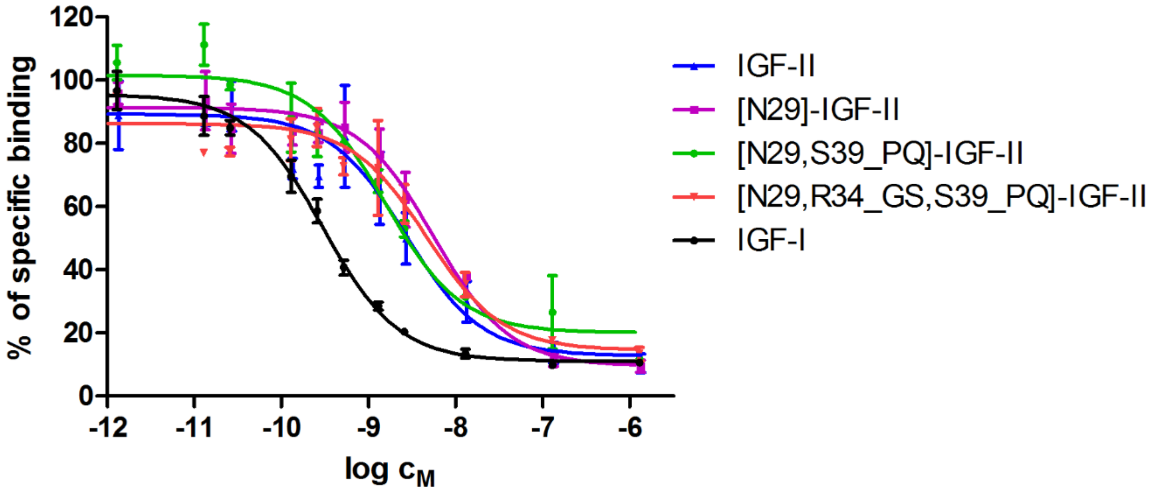


**Figure S5. Inhibition of binding of human [$^{125}$I]-IGF-I to IGF-1R in membranes of mouse fibroblasts by human insulin, IGF-I, IGF-II and IGF-II analogs.**

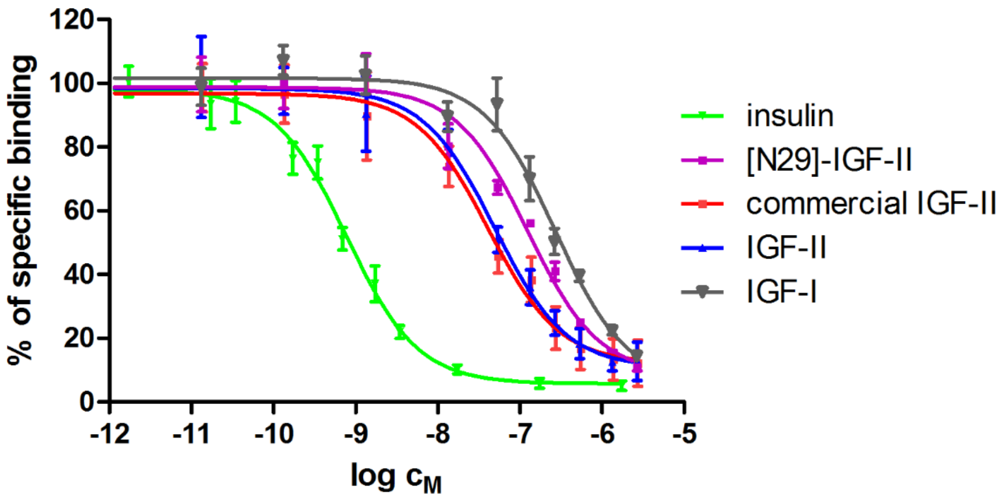


**Figure S6. Inhibition of binding of human [$^{125}$I]-insulin to IR-B in membranes of mouse fibroblasts by human insulin, IGF-I, IGF-II and [N29]-IGF-II analog.**

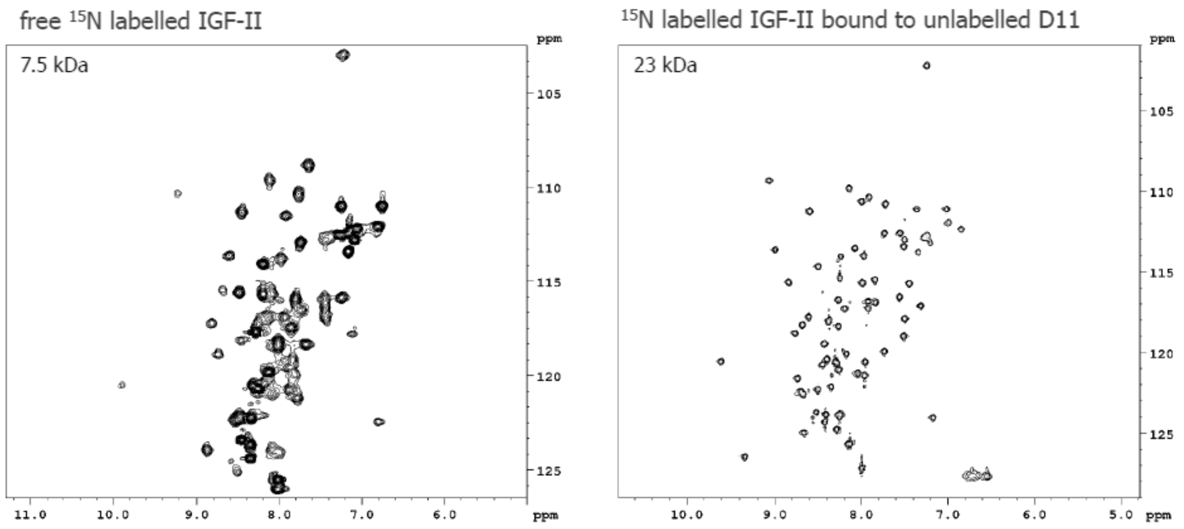


**Figure S7. Significant narrowing of IGF-II signals in $^{1}$H/$^{15}$N HSQC spectrum upon binding to IGF-2R Domain 11.** A spectrum of free $^{15}$N labelled IGF-II is shown on the left panel. Obtained signals do not correspond to the protein mass of 7.5 kDa. The right panel illustrates the signal narrowing observed for IGF-II bound to Domain 11.

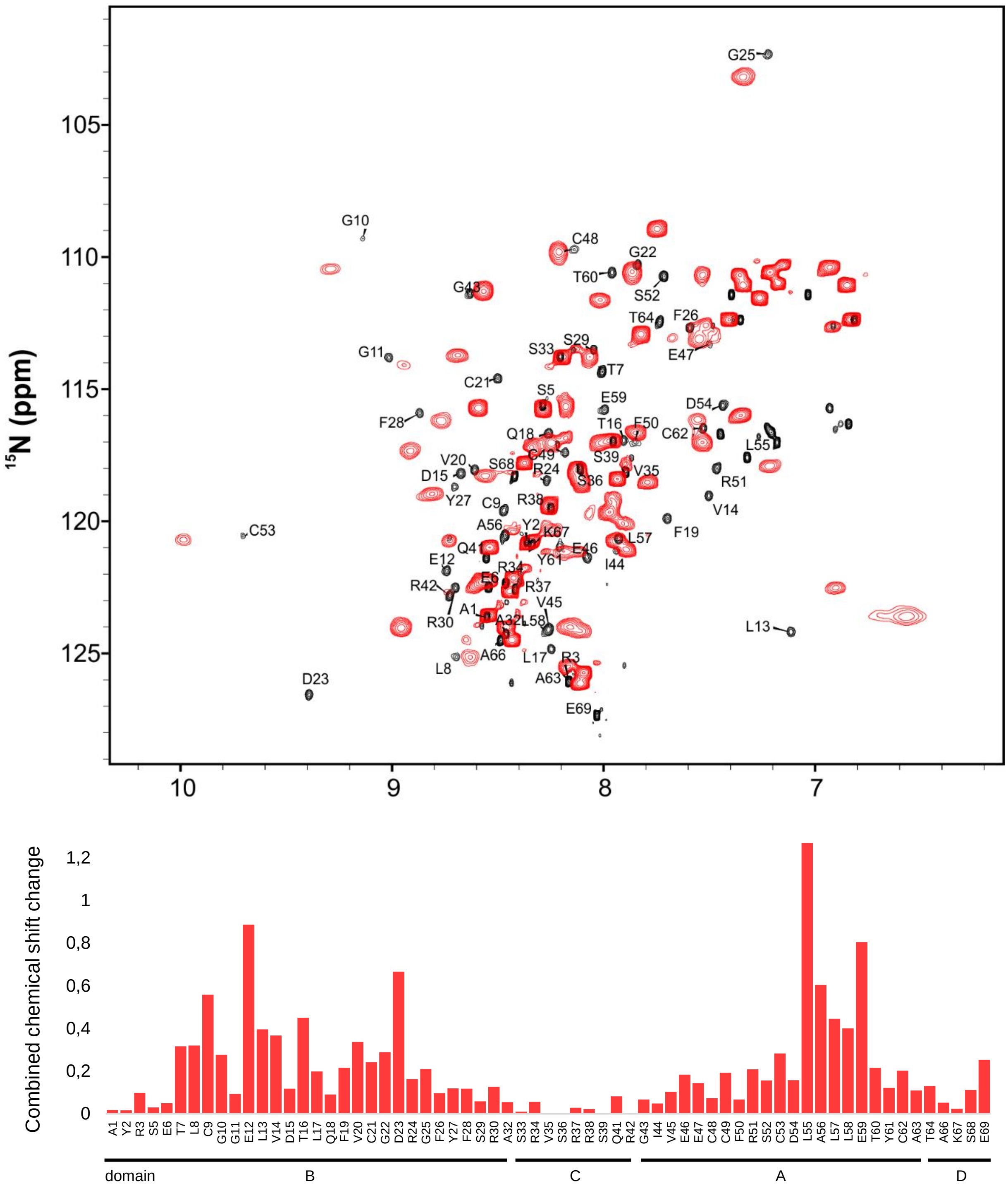


**Figure S8. The C-domain of IGF-II is not affected by D11 binding.**
(**A**) An overlay of $^{1}H/^{15}N$ HSQC spectra obtained for the free (red) and D11-bound [S39_PQ]-IGF-II (black). (**B**) Values of combined chemical shift changes calculated from the changes of backbone amide signal positions. The major differences upon binding to D11 are distributed across the D11 binding interface, while the signals of the C-domain backbone amides bearing the modifications remain relatively unaffected by the D11 binding.

**Table S1.** NMR restraints and structural statistics

| | *IGF-II* | | *[S39_PQ]-IGF-II* | | *[N29, S39_PQ]-IGF-II* | |
|---|---|---|---|---|---|---|
| *Non-redundant distance and angle constrains* | | | | | | |
| Total number of NOE constraints | 1039 | | 1116 | | 1395 | |
| Short-range NOEs | | | | | | |
| Intra-residue (i = j) | 301 | | 315 | | 341 | |
| Sequential (\| i - j \| = 1) | 321 | | 356 | | 406 | |
| Medium-range NOEs (1 < \| i - j \| < 5) | 160 | | 185 | | 281 | |
| Long-range NOEs (\| i - j \| ≥ 5) | 254 | | 257 | | 364 | |
| Torsion angles | 46 | | 46 | | 46 | |
| Hydrogen bond restrains | - | | - | | - | |
| Total number of restricting constraints | 1085 | | 1162 | | 1441 | |
| Total restricting constraints per restrained residue | 16.2 | | 16.8 | | 20.9 | |
| *Residual constraint violations* | | | | | | |
| Distance violations per structure | | | | | | |
| 0.1 – 0.2 Å | 5.05 | | 5.85 | | 9 | |
| 0.2 – 0.5 Å | 2.15 | | 2.3 | | 2.6 | |
| > 0.5 Å | 0 | | 0 | | 0 | |
| r.m.s. of distance violation per constraint | 0.02 Å | | 0.02 Å | | 0.02 Å | |
| Maximum distance violation | 0.45 Å | | 0.48 Å | | 0.48 Å | |
| Dihedral angle violations per structure | | | | | | |
| 1 – 10 ° | 1.3 | | 1.2 | | 1.7 | |
| > 10 ° | 0 | | 0 | | 0 | |
| r.m.s. of dihedral violations per constraint | 0.68 ° | | 0.71 ° | | 0.75 ° | |
| Maximum dihedral angle violation | 5.00 ° | | 5.00 ° | | 5.00 ° | |
| *Ramachandran plot summary from Procheck* | | | | | | |
| Most favoured regions | 94.8% | | 92.2% | | 85.9% | |
| Additionally allowed regions | 5.2% | | 7.8% | | 13.8% | |
| Generously allowed regions | 0.0% | | 0.0% | | 0.1% | |
| Disallowed regions | 0.0% | | 0.0% | | 0.1% | |
| *r.m.s.d. to the mean structure* | *ordered[1]* | *all* | *ordered[1]* | *all* | *ordered[1]* | *all* |
| All backbone atoms | 0.4 Å | 2.9 Å | 1.1 Å | 2.2 Å | 1.0 Å | 1.9 Å |
| All heavy atoms | 1.0 Å | 3.6 Å | 1.7 Å | 2.9 Å | 1.4 Å | 2.5 Å |

[1] Residues with sum of phi and psi order parameters > 1.8
